# Safety of Early Mobilization in Adult Neurocritical Patients: An Exploratory Review

**DOI:** 10.1155/ccrp/4660819

**Published:** 2025-02-25

**Authors:** Leonardo Arzayus-Patiño, José Luis Estela-Zape, Valeria Sanclemente-Cardoza

**Affiliations:** ^1^Faculty of Health, Physiotherapy Program, Universidad Santiago de Cali, Cali, Colombia; ^2^Faculty of Health Sciences, Fundación Universitaria María Cano, Cali, Colombia

## Abstract

**Introduction:** Early mobilization has shown significant benefits in the rehabilitation of critically ill patients, including improved muscle strength, prevention of physical deconditioning, and reduced hospital length of stay. However, its safety in neurocritical patients, such as those with strokes, traumatic brain injuries, and postsurgical brain surgeries, remains uncertain. This study aims to map and examine the available evidence on the safety of early mobilization in adult neurocritical patients.

**Methods:** A scoping review was conducted following PRISMA-SCR guidelines and the Joanna Briggs Institute (JBI) methodology. The research question focused on the safety of early mobilization in neurocritical patients, considering adverse events, neurological changes, hemodynamic changes, and respiratory changes. A comprehensive search was performed in databases such as PubMed, BVS–LILACS, Ovid MEDLINE, and ScienceDirect, using specific search strategies. The selected studies were assessed for methodological quality using JBI tools.

**Results:** Of 1310 identified articles, 25 were included in the review. These studies comprised randomized controlled trials, prospective observational studies, retrospective studies, and pre- and postimplementation intervention studies. The review found that early mobilization in neurocritical patients is generally safe, with a low incidence of severe adverse events, and does not increase the risk of vasospasm, and most complications were manageable with protocol adjustments and continuous monitoring.

**Conclusion:** Early mobilization in neurocritical patients has been shown to be potentially safe under specific conditions, without a significant increase in severe complications when properly monitored. However, the available evidence is limited by the heterogeneity of protocols and study designs, emphasizing the need for further research. The importance of tailoring mobilization protocols to each patient and ensuring continuous monitoring is highlighted. Additional studies with larger sample sizes are needed to fully understand the associated risks and optimize mobilization strategies.

## 1. Introduction

Early mobilization (EM) has been identified as a potentially beneficial intervention in the field of rehabilitation, with positive effects observed in critically ill patients and those with prolonged hospital stays [1]. Among the potential benefits are improved muscle strength [2, 3], prevention of physical deconditioning [4], and an increase in days out of the hospital and intensive care unit [2, 5], which could contribute to better recovery and potentially reduce the economic burden on the healthcare system. Various clinical studies support the implementation of EM, and several clinical practice guidelines have emphasized its safety and effectiveness [6, 7, 8].

These clinical practice guidelines generally focus on hospitalized patients or those in intensive care units with cardiovascular, respiratory, and neurological conditions [6, 7]. While they acknowledge contraindications such as intracranial hypertension, acute intracerebral hemorrhage, and severe cerebral edema, they do not comprehensively address neurocritical patients. This group includes those with strokes (CVA), traumatic brain injuries, and postoperative brain surgeries, who require intensive care due to the complexity of their neurological injuries.

Neurocritical patients present challenges due to the severity of their conditions and the need for specialized management to prevent complications. In particular, those with cerebral edema, aneurysmal or nonaneurysmal hemorrhage, and risk of vasospasm, even if not experiencing intracranial hypertension at the time of intervention, can develop complications requiring continuous monitoring. The management of these patients involves strategies to control and reduce the risk of increased intracranial pressure (ICP), as a significant rise in ICP could exacerbate neurological damage and complicate the recovery process [9–13].

In this group of patients, three main categories are identified: those with hemorrhagic or ischemic stroke, patients with traumatic brain injury (TBI), and those who have undergone brain tumor resection. Patients with aneurysmal subarachnoid hemorrhage (SAH), in particular, present a high risk of complications such as rebleeding and vasospasm, especially between the second and third weeks [14–17]. EM in these cases has been a topic of debate. According to the most recent guideline [18], retrospective studies have shown that early rehabilitation after securing the aneurysm is feasible and safe, with no significant increase in adverse events. However, the guideline also emphasizes that no randomized clinical trials specifically evaluate the safety and effectiveness of this practice in this population.

While EM has been observed to be viable under controlled conditions, questions remain about its impact on specific complications such as vasospasm. Some studies [19] suggest that it could increase the risk, while others indicate that, when conducted appropriately and with adequate monitoring, it could help reduce this risk [14].

The current literature on EM in neurocritical patients is limited and does not provide conclusive guidance on the safety of this practice in this specific group, leading to uncertainty regarding its safety [4]. Although some studies have addressed this issue, the results have been varied and, in many cases, inconclusive [20, 21]. Additionally, mobilization protocols can differ significantly between institutions, further adding to the uncertainty about the safe implementation of these interventions.

Nevertheless, the potential benefits of EM in neurocritical patients warrant consideration. This practice could offer improvements in clinical and functional outcomes for this population, such as enhanced muscle strength, reduced physical deconditioning, and decreased hospital length of stay [20], which could translate into better quality of life and faster recovery for these patients [22–24].

Therefore, it is relevant to map and analyze the available evidence on the safety of EM in neurocritical patients. This leads to the following research question: What is the safety of EM in adult neurocritical patients?

## 2. Methods

This review followed the guidelines of the Preferred Reporting Items for Systematic Reviews and Meta-Analyses Extension for Scoping Reviews (PRISMA-SCR) checklist [25] and was based on the Johanna Briggs Institute (JBI) methodology [26], initially conceived by Arksey and O'Malley [27].

### 2.1. Research Question

To guide the scoping review, the following question was formulated: What is the safety of EM in adult neurocritical patients? The question was designed based on population (divided into three groups), intervention, comparison, and outcomes:

• P (Population): Adult neurocritical patients (patients with acute and subacute ischemic and hemorrhagic stroke, TBI, and postsurgical patients from any brain surgery)
• I (Intervention): Studies applying EM
• C (Comparison): Not applicable
• O (Outcomes): Adverse events, neurological changes, hemodynamic changes, respiratory changes

### 2.2. Selection Criteria

To identify relevant studies, a bibliographic search was conducted based on the research question. Studies were included if they met the following inclusion criteria: primary research, expert recommendations, gray literature, guidelines or protocols, studies published without time restrictions, and publications available in Spanish, English, or Portuguese.

Studies exclusively evaluating passive interventions, such as functional electrical stimulation, and those lacking specific data on active or EM were excluded. These criteria ensured that the selected studies were relevant and directly aligned with the objectives of the analysis.

### 2.3. Sources of Information

Based on the research question, a comprehensive search was conducted in the following electronic databases: PubMed, BVS–LILACS, Ovid MEDLINE, and ScienceDirect. Keywords were determined using Medical Subject Headings (MeSH), Health Sciences Descriptors (DeCS), and natural language.

### 2.4. Search Strategy

Both controlled and uncontrolled language was used, defining terms (MeSH) and (DeCS) with keywords in English, Spanish, and Portuguese. These were used to design specific search equations for each database. Keywords were defined according to the PICO question, allowing the creation of search equations with the main descriptors and qualifiers structured in the Thesaurus. The equations are described in Table 1. The search was manually completed by reviewing the bibliographic references of the found articles using the following updated keywords.

### 2.5. DeCS–MESH

• Neurocritical Care/Critical Care/Intensive Care Units (ICUs)/Brain Injuries/Traumatic Brain Injury (TBI)/Diffuse Axonal Injury/Brain Hemorrhage/Intracranial Hemorrhages/Subarachnoid Hemorrhage/Stroke/Ischemic Stroke/Hemorrhagic Stroke/Intracranial Pressure/Intracranial Hypertension/Neurocritical/critical care/brain injuries/stroke/intracranial pressure
• Rehabilitation/Neurologic Rehabilitation/Early Ambulation/Exercise Therapy/Early Rehabilitation/Physical Therapy Modalities/Mobilization/Early Mobilization/Early Rehabilitation/Physical Therapy/Postural Balance/Postural Control/Muscle Strength/Muscle Weakness
• Patient Safety/Safety Management/Risk Assessment/Adverse Effects/Adverse Events/Complications/Iatrogenic Disease

For the initial search, concepts were standardized in the BVS portal and PubMed databases. Subsequently, an advanced bibliographic search was conducted in the databases listed in Table 1. After reviewing the included articles and consulting additional references, new relevant terms were identified. This allowed for a more specific second search, with the terms and formulas detailed in Table 1.

### 2.6. Selection of Sources of Evidence

The search was conducted by two researchers, with guidance from a university biomedical librarian. The process of identification, review, and eligibility was carried out in consensus among the researchers, with oversight and review by a third member.

### 2.7. Data Extraction

Identified studies were downloaded and uploaded into the RAYAN application. The titles and abstracts of the resulting studies were examined, and duplicates were subsequently removed. A critical reading was conducted, and a descriptive table was created with relevant data. One team member reviewed the relevance and adherence to the criteria, and a third researcher performed quality control by evaluating the excluded articles. There were no discrepancies among the researchers. Tables 2 and 3 describe the characteristics of the studies.

### 2.8. Characteristics of the Interventions

The reviewed interventions included EM techniques encompassing activities such as bed exercises, assisted transfers, progressive ambulation, and active positioning. The intensity of the interventions was analyzed, identifying the most common activities and specifying the prescription in terms of frequency and intensity to provide a more precise description of the characteristics of the interventions performed.

### 2.9. Quality Assessment of the Studies

To evaluate the methodological quality of the selected studies, the Joanna Briggs Institute (JBI) tool [21] was used, with specific checklists for each type of study. Each tool included a numbering system for the evaluated items, and a percentage was calculated based on the total number of items to determine the risk of bias.  Table 4 summarizes the scores obtained.

### 2.10. Definition and Classification of Adverse Events

To comprehensively evaluate the safety of EM, an analysis of the adverse events reported in the selected studies was included. These events were classified based on their severity and the affected system (hemodynamic, respiratory, or neurological), providing a standardized framework for interpretation. The classification was defined as follows:

• *Minor Adverse Events*: Temporary changes that do not require significant medical intervention and resolve spontaneously, such as mild hypotension, fatigue, or transient desaturation that does not require oxygen support.
• *Moderate Adverse Events*: Changes that require healthcare personnel intervention but do not pose an immediate life-threatening risk, such as sustained desaturation requiring oxygen support, controllable hypertension, or hypotension.
• *Severe Adverse Events*: Significant changes that pose a life-threatening risk, require immediate intervention, or result in permanent sequelae, such as severe vasospasm, significant intracranial hypertension, severe arrhythmias, or cardiopulmonary arrest.

Table 5 provides detailed definitions and classifications used.

### 2.11. Presentation of Results

**Figure 1:**
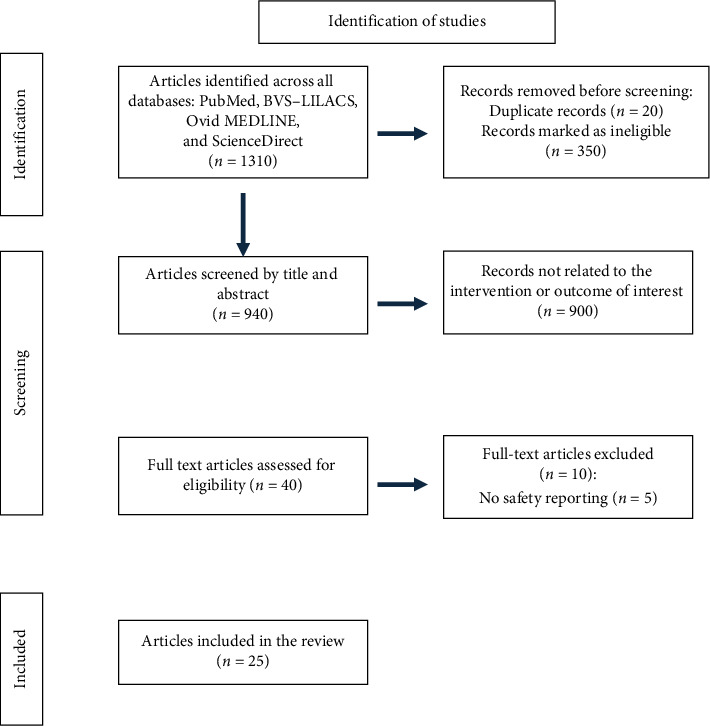
Flowchart of the search process.

The results are presented in descriptive tables highlighting relevant aspects of each study, such as study type, objective, population, type of mobilization, adverse events related to mobilization, and safety. Tables illustrating the search strategy with the equations used (Table 1) and the final number of included studies are presented, along with a flowchart describing the search process and the final number of studies (Figure 1).

## 3. Results

A total of 1310 articles were identified in the databases. After preselection and evaluation by title and abstract, inclusion and exclusion criteria were applied, and nonrelevant articles were excluded. Following this review, 40 articles were selected for full-text reading. Finally, 25 articles were included in this review (Figure 1).

### 3.1. Study Characteristics

Among the identified articles, there were twelve randomized controlled trials, six prospective observational studies, three retrospective studies, two pre- and postimplementation intervention studies, and three prospective intervention studies. Of these studies, seven were conducted in the United States [29–33, 28, 47], three in Norway [14, 34, 35], one in Australia, New Zealand, Malaysia, Singapore, and the United Kingdom (The AVERT Trial Collaboration Group, 2015) [36], two in Taiwan [46, 52], one in Japan [37], one in Switzerland [57], two in Italy [39, 49], one in France [51], one in Denmark [55], one in China [53], and one in India [56].

Table 3 details the main characteristics of the studies that included stroke patients. This table was divided into two population groups: patients whose severity required admission to intensive care units, and those admitted to specialized stroke care units, considered noncritical in some settings.  Table 4 presents the characteristics of studies that included patients with TBI and postsurgical brain surgery patients.

### 3.2. Intervention Characteristics

Regarding the interventions performed, seven studies applied EM in stroke patients in specialized care units [34, 36, 51,52, 54, 56, 57], while eight studies focused on stroke patients admitted to intensive care units [28, 29, 35, 30, 31, 37, 53]. Four studies were conducted on patients with moderate-to-severe TBI [32, 46, 39, 49]. Additionally, one study evaluated an intervention protocol in neurocritical patients with various conditions [46], and another investigated a progressive mobility program in the neurosurgical ICU [38]. Other studies included EM in patients with chronic subdural hematoma [40], gradual mobilization in severe brain injuries [49], and the neurological ICU [35]. Finally, one study evaluated light exercise to reduce cerebral vasospasm [31].

### 3.3. EM

Interventions in patients with stroke (CVA), TBI, and brain surgery exhibit notable heterogeneity in terms of type, frequency, and intensity. The interventions ranged from passive mobilization techniques to progressive active mobilization, with significant differences in initiation times and session durations, depending on the clinical condition of the participants.

In CVA patients, EM was typically initiated within 24–72 h after the event or hospital admission. Common activities included getting out of bed, sitting, standing, and walking. Studies such as the AVERT trial [36] and those conducted by Yen et al. 2020 [52] reported variable intervention frequencies, ranging from multiple daily sessions with durations of 5–30 min. However, these interventions lacked uniform prescriptions.

For TBI patients and those in postsurgical conditions, mobilization strategies included techniques such as assisted passive movements, standing exercises, and progressive walking. The author [46] implemented progressive mobilization within a range of 24–72 h after admission, adjusting activities based on each patient's tolerance. Similarly, Pinto et al. [40] applied EM out of bed following surgery, emphasizing the need to tailor interventions individually. Nonetheless, the duration and intensity of sessions varied considerably across studies.

### 3.4. Safety and Adverse Events in Stroke Patients

#### 3.4.1. Studies in Specialized Stroke Units

Studies conducted in specialized units for the management of stroke evaluated different subgroups of patients, including those with SAH, ischemic stroke, and a combination of both types of stroke.

In the SAH patient group, a specific study [53] reported no adverse events in the intervention group. For patients with ischemic stroke [52, 54, 57], EM was well-tolerated, with no major complications attributed to the intervention [51]. Although minor adverse events, such as falls, were observed, there were no significant differences between groups [54]. Additionally, EM reduced severe complications without affecting cerebral blood flow or neurological outcomes [57].

In studies including patients with both ischemic and hemorrhagic strokes, the AVERT Trial Collaboration (2015) [36] found no significant differences in nonfatal serious adverse events between the EM and usual care groups. Conversely, Sundseth and Thommessen, and Rønning [34] reported similar complications in both groups, although the late mobilization group experienced more events related to immobility. However, they concluded that very EM (VEM) might not be safe, showing a trend toward worse outcomes and higher mortality. EM was considered safe, resulting in better functional status without major complications [56].

### 3.5. Intensive Care Unit

In the group of stroke patients admitted to intensive care units, no adverse events or complications were attributed to EM [29, 30, 38, 54]. Olkowski et al. reported events such as a decrease in mean arterial pressure and an increase in heart rate over 120 beats per minute in 5.9% of the sessions. Karic et al. (2015) [35] documented headaches, fatigue, hypertension, tachycardia, and hydrocephalus, all appropriately managed and not directly attributed to early rehabilitation.

Vasospasm was a major concern in the setting of EM and SAH in several studies. Karic et al. [14] reported vasospasm in 21.6% of patients, which was managed appropriately by allowing mobilization to continue in mild cases and pausing in severe cases. Riordan et al. [31] found that light exercise, including EM, significantly reduced vasospasm frequency and severity, as well as markers of inflammation and oxidative stress. Young et al. [29] documented episodes of hypertension, elevated ICP, and symptomatic vasospasm leading to session interruptions, but no serious adverse events were attributed to EM.

### 3.6. Safety and Adverse Events in TBI and Surgery Patients

In the evaluated studies, EM in patients with TBI and neurosurgery was found to be safe, with manageable adverse events. Pinto et al. [40] and Elkbuli et al. [32] reported significant improvements in mobility and reductions in hospital and ICU stays, without serious adverse events. Rocca et al. [38] observed episodes of hypotension in the standard mobilization group, but mobilization with Erigo was safe and well-tolerated. Yen et al. [46] and Frazzitta et al. [49] also highlighted improvements in functional and neurological outcomes, with no serious adverse events. Bartolo et al. [39] showed significant improvements in patients with severe acquired brain injury, and Bahouth et al. [47] documented minor events such as hypotension, desaturation, and arrhythmias, all properly managed. Jarvis et al. [33] reduced hospital stays, with manageable episodes of hypotension and desaturation.

Riberholt et al. [55] confirmed that early orthostatic exercise in patients with severe TBI was feasible and safe, with a high completion rate of sessions and only minor events, such as orthostatic hypotension and fever, without serious complications.

### 3.7. Risk-of-Bias Assessment (Methodological Quality)

The methodological quality was assessed using the JBI tool [26]. The studies generally showed a low risk of bias with moderate-to-high methodological quality. Table 3 shows the evaluation of the articles.

## 4. Discussion

The objective of this review was to describe and present evidence on the safety of EM in neurocritical patients, finding that the practice results in a low level of adverse events. Most studies report that EM is safe and feasible. The reviewed studies indicated a low incidence of serious adverse events, which were mostly manageable through adjustments in mobilization protocols and continuous monitoring. Additionally, improvements in functional outcomes and a reduction in hospital stay duration were observed, without a significant increase in complications.

The results suggest that EM is safe in stroke patients (CVA) [14, 29, 30, 31, 36, 37, 51, 52, 54, 57], trauma patients [32, 33, 38], and postsurgical patients [40]. Kumar, Romero, and Dharaneeswaran [41] highlighted that EM in critical patients helps reduce ICU stay duration, improve clinical and functional outcomes, and alleviate the economic burden on the healthcare system. Only one study concluded that the VEM group showed a trend toward worse outcomes and higher mortality [34].

The safety of EM in patients with SAH varies depending on the type of bleeding and the presence of aneurysms. In patients with nonaneurysmal SAH, there is a high risk of rebleeding between 48 and 72 h, as reported by Minhas et al. [42]. This risk is considerably high due to the fragility of the vessels and the hemodynamic instability that characterizes these patients. Ma, Wang, and Liu [43] suggest that EM in these cases should be evaluated cautiously due to the significant risk of rebleeding.

In aneurysmal SAH, the reviewed studies do not include patients whose aneurysm has not been secured due to the high risk of rebleeding. This suggests that EM in these cases has not been extensively explored, as the risk may outweigh the potential benefits and increase morbidity and mortality. Dukatz et al. [44] emphasize the need for a cautious approach, recommending initiating EM only after securing the aneurysm.

In contrast, once the aneurysm is secured, EM in patients with aneurysmal SAH is considered feasible and safe. Karic et al. [14] reported the absence of severe adverse effects, although pauses were made due to headaches, fatigue, hypertension, tachycardia, and hydrocephalus, which were managed appropriately. Young et al. [29] demonstrated that EM is safe in patients with SAH and external ventricular drainage, and it was associated with better functionality at discharge. Olkowski et al. [28] concluded that EM is viable, with a low rate of adverse events and no increase in 30-day mortality. Jarvis et al. [33] evidenced that, with adequate monitoring, EM improved mobility and significantly reduced hospital stay. However, it is crucial to monitor blood pressure and ICP, as they can be altered by physical activity and increase the risk of rebleeding.

The safety of EM in patients with SAH has been widely evaluated. Although vasospasm is a common complication in up to 70% of patients, usually occurring between the third and tenth days posthemorrhage [43], studies have not shown that EM increases this risk. Karic et al. [14, 17] reported that EM, initiated from the first day after securing the aneurysm, did not significantly increase adverse events related to vasospasm. Young et al. [29] confirmed that EM in patients with SAH and external ventricular drainage did not increase the incidence of vasospasm, and Riordan et al. [31] observed that light exercise was associated with a reduction in the frequency and severity of vasospasm.

In patients with severe vasospasm, EM should be performed with caution. Karic et al. [14, 35] emphasized that in cases of severe vasospasm, mobilization had to be interrupted until the vasospasm resolved to avoid additional complications. Young et al. [29] found that some mobilization sessions in patients with symptomatic vasospasm also had to be suspended. The evidence suggests that the decision to mobilize should be based on continuous evaluation of the patient's neurological and hemodynamic status, adapting or suspending mobilization in the presence of severe vasospasm to ensure patient safety. This aligns with Hodgson et al. [45], who support that out-of-bed mobilization is contraindicated in the presence of acute neurological changes or when vasopressors are required.

Regarding EM in patients with TBI, it was found to be safe and beneficial with adequate monitoring. Yen et al. [46] reported improvements in mobility without significant adverse events. Elkbuli et al. [32] demonstrated a significant reduction in hospital stay and lower complication rates, without serious adverse events. Similarly, Yen et al. [46] observed improvements in functional mobility without additional harm. However, Rocca et al. [38] indicated that EM is well-tolerated and safe with monitoring devices, although caution should be taken in patients with orthostatic hypotension. Bahouth et al. [47] support the safety of EM in neurocritical patients, including TBI, and emphasize the need for continuous monitoring to manage minor adverse events.

Despite the reported safety, associated risks such as hypotension and increases in ICP should be considered. A decrease in cerebral perfusion pressure can damage recovering ischemic areas. Godoy [48] highlights that to preserve neuronal survival after severe TBI, it is crucial to maintain adequate cerebral perfusion pressure. Although EM can cause variations in this pressure, close monitoring is essential to ensure the patient's hemodynamic stability.

Variations in the type, frequency, and intensity of EM interventions can significantly impact outcomes in neurocritical patients. The observed heterogeneity in the timing of initiation, activities performed, and session duration complicates the generalization of findings across different studies. For instance, the AVERT trial [37] documented an increase in minor adverse events in groups subjected to very early and high-intensity mobilization, suggesting that excessive intensity might be associated with greater risks. Conversely, studies by Karic et al. [14] and Young et al. [29] found that less intensive interventions tailored to the specific conditions of patients were better tolerated and not associated with a significant increase in complications.

The comparison of these variations highlights the importance of designing personalized protocols that take into account the specific clinical conditions of patients. Moreover, studies such as those by Riordan et al. [31] and Olkowski et al. [28] emphasize that a gradual progression, based on patient tolerance, could reduce the incidence of adverse events while optimizing functional benefits. These differences underscore the need to develop standardized protocols that include key parameters such as the timing of initiation, intensity, and duration, enabling more consistent comparisons across studies and yielding more robust and applicable conclusions regarding the safety and efficacy of EM in neurocritical populations.

The safety of EM can be significantly enhanced through the implementation of standardized protocols and systematic tools for risk assessment. The findings of this review highlight that continuous monitoring and the personalization of interventions are essential to minimize adverse events such as hypotension, desaturation, or increased ICP.

The use of safety detection algorithms, such as standardized evaluation scales or presession checklists [45], represents a key strategy to identify specific risk factors before initiating EM. These tools not only allow for precise risk identification but also facilitate risk stratification by classifying patients according to their likelihood of experiencing adverse events. This is particularly crucial in vulnerable populations, such as neurocritical patients, who require stricter monitoring and highly personalized care.

A comprehensive initial assessment should include an integral analysis of the patient's neurological [58], hemodynamic [59], and respiratory status [60]. This involves continuous monitoring of key parameters such as blood pressure, ICP, heart rate, oxygen saturation, and cerebral perfusion pressure levels. The detection of signs of instability, such as altered consciousness, fluctuations in blood pressure, or oxygen saturation, may indicate the need to postpone or adjust mobilization. Additionally, it is essential to identify temporary contraindications before the intervention, such as severe vasospasm, hemodynamic instability [61], high oxygen support requirements [62], fever, or active infections. It is also crucial to assess the use of invasive medical devices, such as catheters, drains, or ICP monitoring systems, to ensure their stability and functionality during EM.

These evaluations must be complemented by the implementation of clear criteria for the progression or suspension of mobilization, based on the patient's tolerance. Any deterioration in monitored parameters or the appearance of symptoms such as pain, dyspnea, or extreme fatigue should signal the need to halt or modify the intervention. This approach could optimize the safety of EM.

The progression of activities should be based on a gradual evaluation of the patient's tolerance, beginning with low-energy movements in bed, followed by activities such as sitting on the edge of the bed, assisted transfers, and, finally, supervised standing and walking with or without assistance. Furthermore, progression must be personalized and adapted to the patient's clinical response, with immediate interruptions or modifications in cases of decompensation.

EM in neurocritical patients requires a multidisciplinary approach [63] to ensure both its safety and efficacy. Physical therapists lead the implementation of progressive physical activities, tailoring them to each patient's clinical conditions. Physicians oversee neurological and hemodynamic stability, adjusting critical parameters to minimize risks. Nurses continuously monitor patients, manage barriers such as catheters and intravenous fluids, and provide support during sessions. Additionally, disciplines such as occupational therapy and nutrition contribute to the process by promoting functionality and optimizing muscle and energy recovery.

The integration of standardized bundles, such as the ABCDEF model in critical care, which incorporates EM and fosters multidisciplinary collaboration, has proven effective in optimizing implementation and reducing secondary complications [64, 65, 66]. Furthermore, adequate collaboration and communication between rehabilitation and medical teams have been suggested to significantly improve outcomes, increase mobilization in this patient population [67], and reduce hospital stays in neurological patients [68]. Interdisciplinary collaboration in the intensive care unit is essential, as constant and efficient communication between the different disciplines ensures that safety measures and medical interventions are maintained during the implementation of EM [69], thereby reducing potential risks.

The methodological quality of the studies was evaluated using the JBI tools. The randomized controlled trials [36, 38, 40, 49, 50, 51–56] demonstrated high methodological quality with scores ranging from 81% to 90%. Other studies presented moderate methodological quality [28, 29, 31, 34, 35, 38, 39, 46, 47, 57] with scores ranging from 70% to 82%. Retrospective and quality improvement studies, such as those by Elkbuli et al. [32] and Jarvis et al. [33], were considered of high quality with a score of 100%. These results demonstrate the validity of this review's findings, as the included studies meet good quality standards in their research.

The review is notable for its methodological strengths, including the rigor in applying standardized protocols and evaluating the quality of the studies. However, several limitations were identified in the present study, particularly regarding the safety of EM. Many studies did not primarily focus on providing detailed documentation of adverse events related to this intervention, which made it difficult to gather accurate and consistent data. Additionally, a considerable number of included studies were retrospective or nonrandomized in design, which can introduce bias and limit the generalizability of the findings. Heterogeneity in EM protocols was also observed, with differences in prescriptions across studies, complicating direct comparisons of results and the development of standardized recommendations. Furthermore, the small sample sizes in some studies provided low statistical power and limited the ability to detect significant differences, underscoring the need for randomized clinical trials to rigorously evaluate this intervention.

## 5. Conclusion

EM in neurocritical patients has been shown to be potentially safe under specific conditions, without a significant increase in severe complications when properly monitored. However, the available evidence is limited by the heterogeneity of protocols and study designs, emphasizing the need for further research. The importance of tailoring mobilization protocols to each patient and ensuring continuous monitoring is highlighted. Additional studies with larger sample sizes are needed to fully understand the associated risks and optimize mobilization strategies.

## Figures and Tables

**Table 1:** Search strategies conducted in each database.

Journal	Number of articles	ST	SA	FR	FS	Question
PubMed	926	21	14	9	9	(“Neurocritical care” OR “neurologic manifestations” OR “critical care” OR “brain injuries” OR “stroke” OR “intracranial pressure” OR “medications and therapies” OR “rehabilitation”
						AND (“patient safety” OR “safety management” OR “risk assessment” OR monitoring, physiologic” OR “equipment safety” OR “motor activity” OR “exercise therapy” OR “rehabilitation” OR “physical therapy modalities”
						AND patient safety OR safety management OR risk assessment OR adverse effects

BVS–LILACS	15	16	3	3	3	(“Critical care” OR “intensive care units” OR “brain injuries” OR “stroke” OR “intracranial pressure”)
						AND (“early ambulation” OR “exercise therapy” OR “rehabilitation” OR “physical therapy modalities” OR “muscle strength” OR “motor activity” OR “postoperative care” OR “critical care” OR “patient positioning” OR “physical fitness” OR “functional recovery” OR “immobilization” OR OR “recovery of function” OR “health status Indicators”)
						AND (“patient safety” OR “safety management” OR “risk assessment” OR “accidental falls” OR “immobilization” OR “moving and lifting patients” OR “protective devices” OR “adverse effects” OR “monitoring, physiologic” OR “equipment safety” OR “workplace safety” OR “health facility environment” OR “restraint, physical”))

ScienceDirect	100	7	4	2	3	((“Critical care” OR “intensive care units” OR “brain injuries” OR “stroke” OR “intracranial pressure” OR “rehabilitation”)
						AND (“early ambulation” OR “exercise therapy” OR “rehabilitation” OR “physical therapy modalities” OR “functional recovery” OR “patient outcome assessment” OR “recovery of function” OR “health status indicators”)
						AND (“patient safety” OR “safety management” OR “risk assessment” OR “accidental falls” OR “moving and lifting patients” OR “protective devices” OR “patient harm” OR “adverse effects” OR “physiologic monitoring” OR “equipment safety” OR “workplace safety” OR “health facility environment” OR “physical restraint”))

Ovid MEDLINE	154	6	4	3	1	(“Critical care” OR “intensive care units” OR “brain injuries” OR “stroke” OR “intracranial pressure”)
						AND (“early ambulation” OR “exercise therapy” OR “rehabilitation” OR “physical therapy modalities” OR “muscle strength” OR “motor activity” OR “patient outcome assessment” OR “bed rest” OR “immobilization” OR “patient care planning” OR “health status Indicators”)
						AND (“patient safety” OR “safety management” OR “risk assessment” OR “accidental falls” OR “protective devices” OR “patient harm” OR “adverse effects “ OR “health facility environment” OR “physical Restraint”)

**Second search**						

PubMed	96	12	5	3	5	(Neurocritical care OR critical care OR intensive care units OR ICUs OR brain injuries OR traumatic brain injury OR TBI OR diffuse axonal injury OR brain hemorrhage OR intracranial hemorrhages OR subarachnoid hemorrhage OR stroke OR ischemic stroke OR hemorrhagic stroke OR intracranial pressure OR intracranial hypertension OR neurocritical OR critical care OR brain injuries OR stroke OR intracranial pressure)
						AND (mobilization OR early mobilization OR postural balance OR postural control OR muscle strength OR muscle weakness)
						AND (patient safety OR safety management OR risk assessment OR adverse effects OR adverse events OR complications OR iatrogenic disease)

BVS–LILACS	4	3	3	3	0	(Neurocritical care OR critical care OR intensive care units OR ICUs OR brain injuries OR traumatic brain injury OR TBI OR diffuse axonal injury OR brain hemorrhage OR intracranial hemorrhages OR subarachnoid hemorrhage OR stroke OR ischemic stroke OR hemorrhagic stroke OR intracranial pressure OR intracranial hypertension OR neurocritical OR critical care OR brain injuries OR stroke OR intracranial pressure)
						AND (mobilization OR early mobilization OR postural balance OR postural control OR muscle strength OR muscle weakness)
						AND (patient safety OR safety management OR risk assessment OR adverse effects OR adverse events OR complications OR iatrogenic disease)

ScienceDirect	12	6	4	4	4	(Neurocritical care OR critical care OR intensive care units OR ICUs OR brain injuries OR traumatic brain injury OR diffuse axonal injury OR brain hemorrhage OR intracranial hemorrhages OR subarachnoid hemorrhage OR stroke OR ischemic stroke OR hemorrhagic stroke OR intracranial pressure OR intracranial hypertension OR neurocritical OR critical care OR brain injuries OR stroke OR intracranial pressure)
						AND (mobilization OR early mobilization OR postural balance OR postural control OR muscle strength OR muscle weakness)
						AND (patient safety OR safety management OR risk assessment OR adverse effects OR adverse events OR complications OR iatrogenic disease)

Ovid MEDLINE	3	1	0	0	0	(Neurocritical care OR critical care OR intensive care units OR ICUs OR brain injuries OR traumatic brain injury OR diffuse axonal injury OR brain hemorrhage OR intracranial hemorrhages OR subarachnoid hemorrhage OR stroke OR ischemic stroke OR hemorrhagic stroke OR intracranial pressure OR intracranial hypertension OR neurocritical OR critical care OR brain injuries OR stroke OR intracranial pressure)
						AND (mobilization OR early mobilization OR postural balance OR postural control OR muscle strength OR muscle weakness)
						AND (patient safety OR safety management OR risk assessment OR adverse effects OR adverse events OR complications OR iatrogenic disease)

*Note:* Authorship: own.

Abbreviations: FR, full-text review; FS, final selection; SA, selected by abstract; ST, selected by title.

**Table 2:** Characteristics of studies in stroke.

Author/year/country	Title of the study and number of references	Objective of the study	Population and sample	Type of study	Variables evaluated	Type of mobilization	Main results	Reported adverse events	Reports on the safety of early mobilization
*Patients in specialized stroke units*									
The AVERT Trial Collaboration Group/2015/Australia, New Zealand, Malaysia, Singapore, Reino Unido [36]	Efficacy and safety of very early mobilization within 24 h of stroke onset (AVERT): A randomized controlled trial Number of references: 25	To compare the effectiveness of very early and frequent mobilization with usual care after a stroke	The study was conducted in 56 stroke units. A total of 2083 patients completed the assessment and three-month follow-up. 1038 were in the early mobilization group, and 1045 were in the usual care group	Single-blind, randomized controlled trial	Modified Rankin scale (mRS), time to walk independently, nonfatal serious adverse events, immobility-related complications, neurological complications	Early mobilization (within the first 24 h) including sitting, standing, and walking	Early mobilization (EM) showed a decrease in the likelihood of achieving a favorable outcome at three months. No significant differences were observed between the groups in terms of recovery or mortality rates at three months	No significant differences were found in nonfatal serious adverse events between the groups	Feasible and generally safe, with no reports of serious adverse events
						*Intensity*: Mild to moderate			
						*Frequency:* Multiple daily sessions			
						*Duration:* 5–30 min per session			
						Activities: Sitting, standing, and walking under supervision			
Sundseth Thommessen, and Rønning/2012/Norway [34]	Outcome after mobilization within 24 hours of acute stroke: A randomized controlled trial Number of references: 38	Compare very early mobilization (VEM) within 24 h of admission with mobilization between 24 and 48 h after a stroke	56 patients after stroke: 27 in the early mobilization group and 29 in the control group	Randomized controlled single-blind trial	Modified Rankin scale (mRS), time to walk independently, nonfatal serious adverse events, immobility-related complications, neurological complications	VEM within 24 h of admission, several times a day until discharge	Trend toward a higher unfavorable outcome, higher mortality rate, and dependence in the EM group. Better neurological improvement in the control group	Similar complications between both groups; immobility-related events were higher in the EM group but without statistical significance	The study suggests that VEM may not be safe, showing a trend toward worse outcomes and higher mortality, but highlights the need for further research for definitive conclusions
						*Intensity:* Adapted to the patient's needs and capabilities. No specific protocol was defined			
						*Frequency:* Several times per day, until hospital discharge			
						*Duration:* The exact duration of each session was not recorded			
Herisson et al./2016/France [51]	Early Sitting in Ischemic Stroke Patients (SEVEL): A randomized controlled trial Number of references: 38	Test the hypothesis that early sitting would be beneficial for stroke outcomes	67 patients with ischemic stroke, randomly assigned to early sitting [63] and progressive sitting groups (75)	Randomized controlled trial	Modified Rankin score, NIHSS, Barthel index, length of hospital stay, medical complications, sitting tolerance	Early out-of-bed sitting within 24 h vs. progressive sitting over 3 days	No significant difference in Rankin score [0–2] at 3 months. Barthel index slightly higher in the early sitting group	One neurological worsening in a patient from the early sitting group	Early sitting was well-tolerated, and no major complications were attributed to mobilization
						*Intensity:* Patients were mobilized to a sitting position within the first 24 h (early protocol) or progressively over three days with initial inclinations of 30°, 45°, 60°, and finally sitting (progressive protocol). The intensity was determined based on patient tolerance, with a minimum recommended time of 15 min and a maximum of 60 min for the first session			
						*Frequency:* Mobilizations were conducted daily, once per day			
						*Duration:* The first session had an average duration of 56.6 ± 41.7 min in the early mobilization group and 83.7 ± 94.7 min in the progressive mobilization group			
Yen et al./2020/Taiwan [52]	Early mobilization of mild-moderate intracerebral hemorrhage patients in a stroke center: A randomized controlled trial Number of references: 60	To investigate the effectiveness of early mobilization (EM) on functional independence in mild–moderate ICH patients	60 patients with mild-to-moderate intracerebral hemorrhage (ICH), randomly assigned to EM [30] and standard early rehabilitation (SER) [30]	Randomized controlled trial	Functional Independence Measure (FIM-motor), Postural Assessment Scale for Stroke Patients (PASS), Functional Ambulation Category (FAC), length of stay	Early out-of-bed mobilization within 24–72 h vs. standard in-bed rehabilitation	The EM group had significantly higher FIM-motor scores at all follow-up points and shorter hospital stays (*p* = 0.004). FAC showed significant improvement in the EM group at 2 and 4 weeks	No adverse events were reported	Early mobilization was safe with no reported adverse events such as falls or line dislodgement
						*Intensity:* Participants in the early mobilization (EM) group performed out-of-bed activities, including unsupported sitting at the edge of the bed, standing with or without support, and stepping while standing. Activities were tailored to the patient's capacity and monitored to ensure safety			
						*Frequency:* Once daily			
						*Duration:* 30 min per session			
Bernhardt et al./2015/Australia [54]	Early mobilization after stroke early adoption but limited evidence Number of references: 40	Evaluate the effects of early mobilization in ischemic stroke patients	2104 patients, randomized into early mobilization (VER) and standard care groups	Randomized controlled trial	Modified Rankin scale (mRS), complications, mortality, length of hospital stay	Early out-of-bed mobilization within the first 24 h vs. standard care (later mobilization) *Intensity*: Early mobilization out of bed, including unsupported sitting on the edge of the bed, standing with or without support, and taking steps while standing. The intensity was adjusted to the individual capacities of the patients *Frequency*: 1 session per day *Duration*: 30 min per session	No significant differences in complications or mortality, but faster functional recovery was observed in the early mobilization group	Some minor adverse events such as falls, with no significant differences between groups	Early mobilization was safe, with no major complications attributed to early mobilization

Chippala and Sharma/2015/India [56]	Effect of very early mobilization on functional status in patients with acute stroke: A single-blind, randomized controlled trial Number of references: 32	To evaluate the effect of very early mobilization on functional status following an acute stroke	86 patients with acute stroke (42 men and 38 women), randomized into the intervention group [43] and the standard care group [43]	Randomized controlled trial (single-blind)	Barthel ADL index (measured on admission, discharge, and at three-month follow-up)	Intensity: Early mobilization out of bed, including unsupported sitting on the edge of the bed, standing with or without support, and taking steps while standing	The intervention group showed significant improvement in Barthel index scores both at discharge and at the three-month follow-up compared with the standard care group	No significant adverse events reported	Early mobilization was safe, resulting in improved functional status with no major complications
						Frequency: Twice a day for seven days			
						Duration: 5 min of standard care once daily for seven days. Additionally, the intervention group performed very early mobilization within 24 h poststroke for 5–30 min			
Diserens et al./2012/Switzerland [57]	Early mobilization out of bed after ischemic stroke reduces severe complications but not cerebral blood flow: A randomized controlled pilot trial Number of references: 32	To evaluate whether early mobilization after ischemic stroke reduces medical complications and is safe regarding neurological function and cerebral blood flow	50 patients with ischemic stroke, NIHSS score > 6 (8 excluded due to early transfer)	Randomized controlled pilot trial	Severe complications, cerebral blood flow, neurological outcomes at 3 months	Early out-of-bed mobilization at 52 h vs. delayed mobilization at 7 days, once a day	The early mobilization group had fewer severe complications (2/25 vs. 8/17, *p* < 0.006). No differences in cerebral blood flow or neurological outcomes were found	No significant adverse events reported	Early mobilization reduced severe complications without affecting cerebral blood flow or neurological outcomes

*Patients in intensive care unit*									
Olkowski et al./2013/EE.UU [28]	Safety and feasibility of an early mobilization program for patients with aneurysmal subarachnoid hemorrhage Number of references: 34	Determine the safety and feasibility of an early mobilization program for patients with aneurysmal subarachnoid hemorrhage (SAH)	25 patients	Retrospective analysis	Neurological and physiological stability, Barthel index, modified Rankin scale, mortality rates, adverse events during sessions	*Intensity*: Progressive early mobilization including functional training and therapeutic exercises in supine, seated, standing, and walking positions. Activities were adjusted to the patient's tolerance	There was no 30-day mortality. Most sessions included out-of-bed activities and walking	Adverse events were reported in 5.9% of sessions, including mean arterial pressure (MAP) < 70 mm Hg or > 120 mm Hg and heart rate > 130 bpm	Early mobilization is safe and feasible for patients with aneurysmal SAH, with a low rate of adverse events
						*Frequency*: Once daily, with an average of 11.4 sessions per patient during hospitalization			
						*Duration*: Between 30 and 60 min			
Young et al./2019/EE.UU [29]	Safety and feasibility of early mobilization in patients with subarachnoid hemorrhage and external ventricular drain Number of references: 41	Evaluate the safety and feasibility of an EM program in patients with aneurysmal subarachnoid hemorrhage (SAH) and external ventricular drainage (EVD)	56 patients: 15 in phase 0 (no mobility), 24 in phase I (therapist-guided mobility), and 17 in phase II (nurse-guided mobility)	Prospective cohort study	Frequency of mobilization, intracranial pressure (ICP), onset of headache, neurological deficit, days of ICU and hospital stay, days on ventilation, tracheostomy rate, discharge disposition	Early mobilization, including out-of-bed activities with the EVD clamped	More frequent mobilization in phase II. Better discharge disposition in phase II, with no significant differences in ICU and hospital stay days between phases	No adverse events were attributable to EM, although there were session terminations due to elevated ICP and symptomatic vasospasm	EM was safe and feasible, with no serious adverse events and a higher frequency of mobilization compared with standard care
						Intensity: Early mobilization out of bed, including sitting at the edge of the bed, standing with or without support, and walking with assistance. The intensity was adjusted to the patient's tolerance			
						Frequency: Once a day, with an average of 7.1 sessions per patient			
						Duration: Sessions lasted up to 3 h, depending on the patient's tolerance			
Karic et al./2016/Norway [35]	Impact of early mobilization and rehabilitation on global functional outcome one year after aneurysmal subarachnoid hemorrhage Number of references: 38	Describe and quantify the content of early rehabilitation adapted for patients with aneurysmal SAH	37 patients with aneurysmal SAH, 25 with mild grade and 12 with severe grade	Prospective observational study	Time spent in rehabilitation, progression in mobilization, clinical status	Early mobilization according to the rehabilitation algorithm	EM was feasible from the first day after securing the aneurysm. Patients with more severe grades received more rehabilitation time	No serious adverse effects related to EM were observed. Vasospasm occurred in 21.6% of patients. Mobilization was paused in severe cases and continued in mild cases. Pauses or reductions in mobilization due to headaches, fatigue, hypertension, tachycardia, and hydrocephalus were not directly attributed to EM	EM was considered safe and feasible from the first day after securing the aneurysm. The study found no evidence that EM caused vasospasms. The frequency of vasospasms was as expected, reinforcing the conclusion that early mobilization does not facilitate the development of vasospasms
						*Intensity:* Progressive mobilization starting from a supine position, progressing to an upright standing position. Activities were individually adjusted based on the patient's clinical condition			
						*Frequency:* Once per day			
						*Duration:* An average of 30–60 min per session in the early rehabilitation group			
Titsworth et al./2012/EE.UU [30].	The effect of increased mobility on morbidity in the neurointensive care unit Number of references: 46	Evaluate the impact of a progressive mobility program on morbidity in patients in the neurosurgical ICU	3291 patients admitted to the ICU before and after the implementation of the program, with a variety of neurosurgical conditions	Retrospective cohort study of 86 patients, 43 in the EM group and 43 in the control group	Morbidity, duration of ICU stay, days on ventilation, hospital-acquired infections, immobilization rate	Progressive mobility program including 11 steps, from head-of-bed elevation to unassisted ambulation	Significant reduction in immobilization days, hospital-acquired infections, and ICU stay duration. Morbidity was significantly reduced in the EM group	No serious adverse events related to early mobilization were reported	EM was considered safe, with no increase in adverse complications attributable to the program
Karic et al./2017/Norway [14]	Effect of early mobilization and rehabilitation on complications in aneurysmal subarachnoid hemorrhage Number of references: 24	Evaluate the effect of EM on complications during the acute phase and within 90 days postaneurysmal subarachnoid hemorrhage (SAH)	171 stroke patients, 94 in the EM group and 77 in the control group	Prospective intervention study	Frequency and severity of cerebral vasospasm, cerebral infarction, acute and chronic hydrocephalus, pulmonary infections, thromboembolic events, mortality	EM, starting on the first day after aneurysm repair	EM did not increase complications. The frequency and severity of cerebral vasospasm were lower in the EM group	No serious adverse effects related to early mobilization were observed	Early mobilization was safe, reducing the frequency and severity of cerebral vasospasm
						*Intensity:* Progressive early mobilization			
						*Frequency:* Daily			
						*Duration:* The exact duration per session is not specified			
Riordan et al./2015/EE.UU [31]	Mild exercise reduces cerebral vasospasm after aneurysm subarachnoid hemorrhage: A retrospective clinical study and correlation with laboratory investigation Number of references: 24	Evaluate whether light exercise reduces cerebral vasospasm after aneurysmal subarachnoid hemorrhage (aSAH)	20 patients with aSAH in ICU, after aneurysm repair, control group consisted of patients who did not perform light exercise	Retrospective study with laboratory correlation	Frequency and severity of cerebral vasospasm, neurological outcomes, laboratory analysis of inflammatory markers, and oxidative stress	Light exercise, including early mobilization and moderate physical activity	Light exercise was associated with a significant reduction in the frequency and severity of cerebral vasospasm. Laboratory analyses showed a decrease in inflammatory markers and oxidative stress	No serious adverse events related to light exercise were reported	Light exercise was considered safe and beneficial for reducing cerebral vasospasm without increasing complications
Takara et al./2021/Japan [37]	Association between early mobilization and functional outcomes in patients with aneurysmal subarachnoid hemorrhage: A multicenter retrospective propensity score-matched study Number of references: 49	Investigate the association between EM and function in patients with aneurysmal subarachnoid hemorrhage (aSAH)	200 patients with aSAH distribution: 100 patients in the EM group and 100 in the control group	Multicenter retrospective study	Functional outcomes, complication rate, mortality	Mobilization within 24 h after aneurysm repair included getting out of bed and walking	Early mobilization was associated with better functional outcomes and a higher rate of discharge to home	No serious adverse events related to early mobilization were reported	Considered safe and beneficial for improving functional outcomes without increasing complications
						*Intensity:* Progressive mobilization, including activities such as sitting, standing with or without support, and walking with or without assistance, adjusted according to the patient's clinical stability and tolerance			
						*Frequency:* Approximately five times per week			
						*Duration:* Each physical or occupational therapy session lasted at least 20 min			
						*Activities:* Included positioning, range of motion exercises, strength training, activities of daily living training, and supervised walking with or without assistive devices			
Liu et al./2014/China [53]	Randomized controlled trial of early rehabilitation after intracerebral hemorrhage stroke: Difference in outcomes within 6 months of stroke Number of references: 28	Compare early rehabilitation with standard care in patients with intracerebral hemorrhage (ICH)	243 patients with ICH, randomized into very early rehabilitation (VER) (122) and standard care (121)	Randomized controlled trial	Survival, health-related quality of life (SF-36), modified Barthel index, anxiety level (Zung Self-Rated Anxiety Scale), length of hospital stay	*Intensity:* Progressive early mobilization adapted to individual capacities	Patients in the VER group showed higher survival rates at 6 months, with better scores in quality of life and functionality in daily activities	No significant adverse events were reported	Early rehabilitation was safe, with a lower incidence of medical complications such as infections or pulmonary embolisms
						*Frequency:* Conducted at least 16 times per month			
						*Duration:* Each session lasted 60 min			
						*Activities:* Included bed rest or sitting in a chair during the first week of admission in the standard care group. In the VER group, rehabilitation began within the first 48 h, while in the standard care group, it started after 7 days. Activities included functional training and progressive mobilization			

**Table 3:** Traumatic brain injury (TBI) and various brain surgery conditions.

Author/year/country	Title of the study and number of references	Objective of the study	Population and sample	Type of study	Variables evaluated	Type of mobilization	Main results	Reported adverse events	Reports on the safety of early mobilization
Pinto et al./2023/Portugal [40]	GET-UP Trial 1-year results: Long-term impact of an early mobilization protocol on functional performance after surgery for chronic subdural hematoma Number of references: 27	Evaluate the impact of early mobilization on long-term functionality after surgery for chronic subdural hematoma (cSDH)	100 patients with chronic subdural hematoma (cSDH) Distribution: 50 patients in the early mobilization group and 50 in the control group	Randomized clinical trial	Functional outcomes (GOSE and mRS), surgical recurrence	Early postoperative mobilization in patients with cSDH	Significant improvement in long-term functional outcomes without an increase in surgical recurrence	No serious adverse events related to early mobilization were reported Observations: Some patients experienced intolerance to mobilization, but these events were manageable and did not result in severe complications	Early mobilization was considered safe and preferable compared with mandatory bed rest strategies. It improved long-term functional outcomes without increasing surgical recurrence or serious adverse events
						Intervention: Early mobilization out of bed and specific activities as soon as possible, typically within 24 h postsurgery			
						*Duration:* Not specified			
						Control group			
						Intervention: Mandatory bed rest for at least 48 h postsurgery			

Elkbuli et al./2022/EE.UU [32]	The association between early versus late physical therapy initiation and outcomes of trauma patients with and without traumatic brain injuries Number of references: 47	Evaluate the association between early vs. late initiation of physical therapy and outcomes in patients with and without traumatic brain injuries	11,937 patients with TBI, divided into groups with early and late initiation of physical therapy	Retrospective cohort study	Duration of hospital and ICU stay, complications, functional outcomes	Early and late mobilization in trauma patients	Early mobilization was associated with a significant reduction in hospital and ICU stay and lower complication rates compared with late mobilization: 12 days vs. 16 days on average (*p* < 0.001)	No significant serious adverse events related to early mobilization were reported; some episodes of hypotension and fatigue were manageable with adjustments to the mobilization protocol	Considered safe and beneficial for improving functional outcomes and reducing complications
						Experimental group (early mobilization)			
						Intervention: Initiation of physical therapy within the first 5 days posttrauma			
						Includes range of motion exercises, standing activities, and walking, based on patient tolerance			
						Control group (late mobilization)			
						Intervention: Initiation of physical therapy after the first 5 days posttrauma			
						Mobilization based on the hospital's standard protocol			

Rocca et al., 2016, Suiza [38] 2016, Suiza [31–38]	Sympathetic activity and early mobilization in patients in intensive and intermediate care with severe brain injuries: A preliminary prospective randomized study Number of references: 27	Observe and quantify blood pressure changes during gradual mobilization in patients with severe brain injuries	30 patients divided into three groups with severe brain injuries, with a bed rest period of at least 7 days before mobilization	Prospective randomized study	Sympathetic activity (catecholamines), blood pressure	Standard mobilization, MOTOmed, Erigo	Mobilization with Erigo did not increase catecholamine production, unlike standard mobilization and MOTOmed. Blood pressure showed no significant differences between the groups	No significant adverse events were reported. However, there was a trend toward a higher prevalence of hypotensive events in the standard mobilization group	The mobilization with Erigo is safe and well-tolerated, whereas MOTOmed should be used with caution in patients with SAH due to the potential risk of hypotension
						Group 1: Standard mobilization intervention: mobilization performed by physiotherapists, including position changes in bed and assisted active movements			
						Group 2: MOTOmed intervention: mobilization with a lower limb ergometer allowing passive, active, or assisted leg movements			
						Group 3: Erigo intervention: mobilization with an Erigo tilt table allowing progressive verticalization of the patient with leg movements			

Yen et al., 2022, Taiwán [46] 2022, Taiwán [39]	Functional mobility effects of progressive early mobilization protocol on people with moderate-to-severe traumatic brain injury: A pre–post intervention study Number of references: 32	Evaluate the effects of an early mobilization protocol on the functionality of individuals with moderate-to-severe traumatic brain injury	50 patients with moderate-to-severe traumatic brain injury, assessed before and after the intervention	Pre–postintervention study	Functional mobility, independence in daily activities, overall recovery	Early progressive mobilization, including bed exercises, transfers, and assisted walking	Significant improvements in functional mobility and independence in daily activities compared with the preintervention phase	No serious adverse events related to the intervention were reported. Some patients experienced fatigue and episodes of hypotension, which were manageable with protocol adjustments	Considered safe and effective for improving functional mobility and independence in patients with moderate-to-severe TBI
						*Intensity:* Progressive EM protocol tailored to the patient's clinical condition			
						*Frequency:* Early mobilization began within the first seven days of ICU admission and continued daily throughout the patient's stay in the unit			
						*Duration:* Not specified for individual sessions			
						*Activities:* The intervention included progressive activities such as sitting on the edge of the bed and performing functional mobility exercises aimed at improving sensorimotor function and increasing out-of-bed mobility			

Bartolo et al., 2017, Italy [39] 2017, Italy [32–39]	Mobilization in early rehabilitation in intensive care unit patients with severe acquired brain injury: An observational study Number of references: 32	Evaluate whether early mobilization of patients with severe acquired brain injury (sABI) influences functional outcomes	103 patients with sABI, divided into mobilization [68] and nonmobilization [35] groups	Prospective observational study	Glasgow Coma Scale, Disability Rating Scale, Levels of Cognitive Functioning Scale, Barthel index	Early passive and active-assisted mobilization, including postural changes (≥ 6–8 times/day), sitting on the edge of the bed, sitting in a chair, using a tilt bed/table ≥ 40°, and exercises with physical or mechanical assistance to complete the activities	Significant improvements in cognitive FIM, GOS, and ERBI in the mobilization group; higher discharge rates to intensive rehabilitation	No adverse events were reported in either group	Early mobilization is safe and promotes clinical and functional recovery
						*Duration:* Mobilization sessions were defined as continuous periods of mobilization followed by bed rest. The exact duration of individual sessions was not specified			

Bahouth et al., 2018, EE.UU [47]. 2018, EE.UU [40–47]	Safety and feasibility of a neuroscience critical care program to mobilize patients with primary intracerebral hemorrhage Number of references: 28	Evaluate the safety and feasibility of an early mobilization protocol in neurocritical care	60 patients in neurocritical care with various neurological conditions	Prospective observational study	Safety, feasibility, adverse events, functional outcomes	*Early mobilization* within the first 48 h after ICU admission. Includes passive and assisted active mobilization, range of motion exercises, sitting on the edge of the bed, and assisted walking. Once daily for at least 30 min	Early mobilization is safe and feasible and improves functional outcomes: Early mobilization was well-tolerated, with minor adverse events	Events reported included hypotension (10% of patients), desaturation (8%), and arrhythmias (5%). All were managed through adjustments in the mobilization protocol and continuous monitoring	Early mobilization is safe with appropriate monitoring

Jarvis et al., 2023, EE.UU [33]. 2023, EE.UU [25–33]	Implementation of an early mobility protocol in the neuroscience ICU Number of references: 17	Develop and implement an early mobilization protocol in the neurological ICU	198 medical records reviewed: 70 preimplementation and 128 postimplementation	Pre- and postimplementation quality improvement study	Time from admission to first out-of-bed activity, number of documented out-of-bed activities, length of hospital stay	*Intensity:* Early mobilization included passive and active-assisted exercises, posture changes, sitting at the edge of the bed, and assisted walking. A progressive protocol adapted to the patient's clinical needs was utilized, using the Bedside Mobility Assessment Tool (BMAT)	Reduction in the mean time from admission to the first out-of-bed activity from 3.24 to 2.01 days (*p* = 0.06). Significant decrease in hospital length of stay from 19.8 to 12.42 days (*p* = 0.006)	Hypotension, oxygen desaturation, and arrhythmias were documented, all manageable with protocol adjustments and continuous monitoring	Considered safe with appropriate monitoring and adjustments based on the patient's response
						*Frequency:* Approximately five times per week			
						*Duration:* The exact duration of each session is not specified			
						*Activities:* Included mobility assessments, identification of barriers, development of a personalized mobility plan, and execution of the plan			
						*Session duration:* Each session lasted an average of at least 30 min			

Frazzitta et al. (2016)/Italy [49] (2016)/Italy [42–49]	Effectiveness of a very early stepping verticalization protocol in severe acquired brain injured patients: A randomized pilot study in ICU Number of references: 37	Evaluate the effectiveness of a very early stepping verticalization protocol in severe ABI patients	40 patients with vegetative state or minimally conscious state (VS/MCS) post-ABI	Randomized pilot study	Glasgow Coma Scale (GCS), Coma Recovery Scale Revised (CRSr), Disability Rating Scale (DRS), Levels of Cognitive Functioning (LCF)	Very early stepping verticalization using a tilt table with a robotic stepping device	Significant improvement in functional and neurological outcomes. CRSr improvement was better in the verticalization group	None reported	Safe and feasible, with no adverse events reported during the protocol

Yen et al./2024/Taiwan [50]/2024/Taiwan [43–50]	Assessing the impact of early progressive mobilization on moderate-to-severe traumatic brain injury: A randomized controlled trial Number of references: 60	Evaluate the impact of early progressive mobilization (EPM) on patients with moderate-to-severe TBI	65 patients with moderate-to-severe TBI, randomly assigned to EPM group [33] and EPUP group [32]	Randomized controlled trial	Perme ICU Mobility Score, FIM-motor, phase angle (PhA), skeletal muscle index (SMI), length of ICU stay, ventilation duration	Intensity: An early mobilization (EM) protocol was used, including out-of-bed activities as soon as possible, progressing from in-bed exercises to out-of-bed activities based on the patient's tolerance	The EPM group showed higher mobility and shorter ICU stays, with reduced ventilation duration. No significant differences in PhA	None reported	Early mobilization was safe, with no reported adverse events such as falls or tube disconnections
						Frequency: Interventions were performed five times per week			
						Duration: Each session lasted 30 min			
						Activities: Initial activities included head elevation to more than 60°, sitting on the edge of the bed, performing balance exercises, and walking			

Riberholt et al./2021/Denmark [55]/2021/Denmark [48–55]	Early orthostatic exercise by head-up tilt with stepping vs. standard care after severe traumatic brain injury is feasible Number of references: 30	Investigate the feasibility and safety of early orthostatic exercise (head-up tilt with stepping) compared with standard care in severe traumatic brain injury patients	38 patients with severe traumatic brain injury, randomized to early orthostatic exercise [19] and standard care [19]	Randomized controlled trial	Number of included participants, number of conducted mobilization sessions, adverse events, serious adverse events, Coma Recovery Scale, Early Functional Ability, Functional Independence Measure	Head-up tilt with stepping on a tilt table vs. standard care (no regular mobilization on a tilt table)	Early orthostatic exercise was feasible with 74% of intended sessions completed. No significant differences in serious adverse events or adverse reactions between groups	Minor adverse events such as orthostatic hypotension and fever were reported in both groups	Early mobilization was safe with no major complications reported. Further studies are needed to determine clinical benefits

**Table 4:** Quality assessment of studies.

Author	Study type	Quality assessment (tool evaluation) (%)	Interpretation
The AVERT Trial Collaboration Group (2015) [36]	Randomized controlled trial	Joanna Briggs Institute, 85	High quality
Sundseth, Thommessen, and Rønning (2012) [34]	Randomized controlled trial	Joanna Briggs Institute, 76	Moderate quality
Olkowski et al. (2013) [28]	Retrospective study	Joanna Briggs Institute, 82	Moderate quality
Young et al. (2019) [29]	Prospective observational cohort study with historical cohort	Joanna Briggs Institute, 78	Moderate quality
Karic et al. (2016) [35]	Prospective observational study	Joanna Briggs institute, 81	Moderate quality
Kinoshita et al. (2021) [30]	Multicenter retrospective study	Joanna Briggs Institute, 87	High quality
Karic et al. (2017) [14]	Prospective intervention study	Joanna Briggs Institute: 81	High quality
Riordan et al. (2015) [31]	Retrospective study with laboratory correlation	Joanna Briggs Institute: 77	Moderate quality
Titsworth et al. (2012) [30]	Prospective cohort study	Joanna Briggs Institute: 88	High quality
Pinto et al. (2023) [40]	Randomized controlled trial	Joanna Briggs Institute: 90	High quality
Elkbuli et al. (2021) [32]	Retrospective cohort study	Joanna Briggs Institute: 100	High quality
Rocca et al. (2016) [38]	Prospective randomized study	Joanna Briggs Institute: 76	Moderate quality
Yen et al. (2022) [46]	Pre–postintervention study	Joanna Briggs Institute: 70	Moderate quality
Bartolo et al. (2017) [39]	Prospective observational study	Joanna Briggs Institute: 72	Moderate quality
Bahouth et al. (2018) [47]	Prospective observational study	Joanna Briggs Institute: 78	Moderate quality
Jarvis et al. (2023) [33]	Pre- and postimplementation quality improvement study	Joanna Briggs Institute: 100	High quality
Frazzitta et al. (2016) [49]	Randomized pilot study	Joanna Briggs Institute: 84.6	High quality
Yen et al. (2024) [50]	Randomized controlled trial	Joanna Briggs Institute tool: 82	High quality
Herisson et al. (2016) [51]	Randomized controlled trial	Joanna Briggs Institute tool: 80	High quality
Takara et al. (2021) [37]	Multicenter retrospective study	Joanna Briggs Institute tool: 85	High quality
Liu et al. (2014) [53]	Randomized controlled trial	Joanna Briggs Institute tool: 80	High quality
Bernhardt et al. (2015) [54]	Randomized controlled trial	Joanna Briggs Institute tool: 82	High quality
Riberholt et al. (2021) [55]	Randomized controlled trial	Joanna Briggs Institute tool: 78	High quality
Chippala and Sharma. (2015) [56]	Randomized controlled trial (single-blind)	Joanna Briggs Institute tool: 85	High quality
Diserens et al. (2011) [57]	Randomized controlled pilot trial	Joanna Briggs Institute tool: 78	Moderate quality

**Table 5:** Definition and classification of adverse events.

System	Adverse event	Definition	Classification
Hemodynamic	Hypotension	Mean arterial pressure < 60 mmHg or sustained drop > 20% from baseline, managed with fluids or medications	Moderate or severe
Hemodynamic	Hypertension	Sustained mean arterial pressure > 120 mmHg, managed with antihypertensives or protocol adjustment	Moderate
Hemodynamic	Hypertension	Heart rate > 130 bpm sustained, managed with rest or adjustment in mobilization	Moderate
Respiratory	Desaturation	Oxygen saturation < 90% sustained for > 60 s, managed with supplemental oxygen	Moderate or severe
Respiratory	Respiratory arrest	Cessation of spontaneous breathing requiring immediate intubation or ventilatory support	Severe
Neurological	Vasospasm	Clinical and imaging confirmation of significant narrowing in intracranial arteries	Moderate or severe
Neurological	Acute neurological changes	Sudden alteration in consciousness, hemiparesis, or new focal neurological symptoms	Severe
Neurological	Intracranial hypertension	Intracranial pressure > 20 mmHg confirmed by invasive monitoring, managed with sedation or additional interventions	Severe
Systemic	Fatigue	Subjective sensation of exhaustion limiting the ability to continue the mobilization session	Minor

## Data Availability

The data generated and analyzed during this study are available upon reasonable request directed to the corresponding author.
