# Corrigendum to “Safety of Early Mobilization in Adult Neurocritical Patients: An Exploratory Review”

**DOI:** 10.1155/ccrp/9757345

**Published:** 2025-09-16

L. Arzayus-Patiño, J. L. Estela-Zape, and V. Sanclemente-Cardoza, “Safety of Early Mobilization in Adult Neurocritical Patients: An Exploratory Review,” *Critical Care Research and Practice* 2025 (2025): 4660819, https://doi.org/10.1155/ccrp/4660819.

In the above mentioned article, the contact details for Leonardo Arzayus-Patiño were incorrect. The correct contact information is shown below:

Correspondence should be addressed to Leonardo Arzayus-Patiño; leonardoarzayus00@usc.edu.co.

Also, there was an error in the Funding statement, where the institution name and the funding ID number were provided incorrectly as “This research was funded by the Dirección general de investigaciones de Universidad Santiago de Cali, under Call No. 01-2025.” The corrected Funding statement appears below:

**Funding**

This research has been funded by Dirección General de Investigaciones of Universidad Santiago de Cali under Call No. DGI-01-2025.

The corrections are updated in the online article.

We apologize for this error.

